# Feasibility and experience of using exception from informed consent in a pilot study of immediate estrogen infusion for hypotensive trauma patients

**DOI:** 10.1186/cc12226

**Published:** 2013-03-19

**Authors:** JG Wigginton, PE Pepe, V Warren, K AbdelFattah, JW Gaston, J Simpkins, JP Minei, D Maass, AH Idris

**Affiliations:** 1 University of Texas Southwestern Medical Center, Dallas, TX, USA; 2 University of North Texas, Fort Worth, TX, USA

## Introduction

Many proposed resuscitative therapies for cardiac arrest and trauma will require the earliest possible intervention and would occur under volatile circumstances, making true informed consent for clinical trials unfeasible. The purpose here was to report our experience using exception to informed consent during the inaugural pilot study of infusing estrogen for acute injury, the so-called RESCUE Shock study.

## Methods

Fifty patients were enrolled in RESCUE Shock in which estrogen or placebo was infused as soon as possible in the emergency department for trauma patients with a low systolic blood pressure (<90 mmHg) at two level I trauma centers. They were all treated with a single-dose estrogen or placebo infusion within 2 hours using exception from informed consent following US federal guidelines.

## Results

Investigator-initiated exception from informed consent studies is feasible, with our FDA IND approval obtained in 31 days, IRB 1 approval in 25 days, and IRB 2 approval in 24 days. Community consultation/notification was successfully accomplished with no one opting out and 47/50 enrolled patients or their legal representatives were notified of participation (one died unidentified, two died with no known contact). The average number of days to verbal notification of patients or advocates was 6.55 days (range 0 to 51 days) as the study team began notification only after the patient or family was able to reasonably understand information about the study. No one decided against continued follow-up. Overall, patients and their families were very enthusiastic about participation and the data safety monitoring board had no safety concerns after reviewing all study data.

## Conclusion

Although delayed notice of participation occurs for many justifiable reasons, the use of exception from informed consent for novel, time-sensitive resuscitation studies is not only crucial, but can be feasible, and well accepted by patients, their advocates and communities at large.

